# Nitrogen-Doped Carbon Dots as A New Substrate for Sensitive Glucose Determination

**DOI:** 10.3390/s16050630

**Published:** 2016-05-04

**Authors:** Hanxu Ji, Feng Zhou, Jiangjiang Gu, Chen Shu, Kai Xi, Xudong Jia

**Affiliations:** 1 State Key Laboratory of Coordination Chemistry, Department of Polymer Science & Engineering, Nanjing National Laboratory of Microstructures, Nanjing University, Nanjing 210023, China; com_jihanxv@163.com (H.J.); zhoufeng819@163.com (F.Z.); gjjdgqf@gmail.com (J.G.); gandalf404@126.com (C.S.); 2 Department of Polymer Science & Engineering, Nanjing University, Nanjing 210023, China; xikai@nju.edu.cn

**Keywords:** nitrogen-doped carbon dot, glucose oxidase, enzyme immobilization, biosensor, electrochemical detection

## Abstract

Nitrogen-doped carbon dots are introduced as a novel substrate suitable for enzyme immobilization in electrochemical detection metods. Nitrogen-doped carbon dots are easily synthesised from polyacrylamide in just one step. With the help of the amino group on chitosan, glucose oxidase is immobilized on nitrogen-doped carbon dots-modified carbon glassy electrodes by amino-carboxyl reactions. The nitrogen-induced charge delocalization at nitrogen-doped carbon dots can enhance the electrocatalytic activity toward the reduction of O_2_. The specific amino-carboxyl reaction provides strong and stable immobilization of GO_x_ on electrodes. The developed biosensor responds efficiently to the presence of glucose in serum samples over the concentration range from 1 to 12 mM with a detection limit of 0.25 mM. This novel biosensor has good reproducibility and stability, and is highly selective for glucose determination under physiological conditions. These results indicate that N-doped quantum dots represent a novel candidate material for the construction of electrochemical biosensors.

## 1. Introduction

Carbon dots (CDs), a relatively new member of the carbon nanomaterial family, were first obtained during purification of single-walled carbon nanotubes in 2004 [1]. They are generally oxygenous carbon nanoparticles with a size of less than 10 nm. CDs have subsequently attracted considerable attention due to their simple synthesis procedure combined with fascinating physical properties [2]. Just like heavy-metal-based quantum dots (QDs), they exhibit several promising advantages over organic fluorescence dyes, such as tunable luminescence emission, high stability against photobleaching and blinking. In addition, CDs are biocompatible and small dots with low molecular weight and low toxicity, which makes them superior to metal quantum dots [3,4,5]. Applications of CDs in cellular bioimaging, drug delivery and sensing based on their optical characteristics have been successfully demonstrated by several groups [6,7,8,9,10,11]. Zhu and co-workers reported a facile and high-output method for the fabrication of CDs with a quantum yield as high as 80%. CDs have been applied both as printing inks and detection of Fe^3+^ in biosystems [12]. Yu and co-workers displayed a new type of CDs which could form a fluorescence resonance energy transfer (FRET) system with an organic dye. This FRET system could serve as a ratiometric sensor for H_2_S in aqueous solution, biological fluids and living cells [13]. However, most of the applications of CDs are based on their optical characteristics. Electrochemical applications of the CDs are underreported, especially in the detection of biomolecules such as glucose.

On the other hand, a simple, fast, and long-term stationary method for glucose detection with high sensitivity and selectivity is a great demand in a variety of fields ranging from biomedical applications to ecological approaches [14]. Glucose biosensors, which utilize immobilized enzymes for the conversion of the target analytes into electrochemically detectable products, have been one of the most widely used electrochemical detection methods up to now [15]. Especially, screen printed electrodes (SPEs) have been widely used to fabricate disposable and economical electrochemical sensors, which has helped establish the route from ‘lab-to-market’ for a plethora of sensors [16]. For its high bioactivity and stability as well as relatively low in price, glucose oxidase (GO_x_) has been commonly used in glucose biosensors [17]. With the selectivity and sensitivity provided by GO_x_, the remaining most important goal is to make the whole detection process simple, fast, and cheap. For this purpose, different approaches have been developed, among which the use of functional nanomaterials has attracted great attention because nanomaterials can efficiently facilitate electron transfer between enzymes and electrodes, while allowing the detection to occur at low potential. A lot of nanomaterials, such as gold nanoparticles, graphene, carbon nanotubes, *etc.* have been widely used in the fabrication of glucose biosensors [18,19,20,21,22,23,24,25,26,27,28,29,30]. Nitrogen-doped (N-doped) carbon materials in particular have emerged as a powerful tool in glucose enzymatic biosensor development. N-doped graphene and carbon nanotubes have been successfully developed in glucose sensing [31,32]. However, most of these N-doped materials need to dope the N on the pre-synthesis carbon material, which makes the synthesis procedure time-consuming and increases the cost of the whole procedure. As a new carbon material, N-doped CDs have not been widely used yet in electrochemical biosensors. Compared with other N-doped carbon materials, it is easier and more convenient to dope the nitrogen on the carbon materials by using a nitrogen-containing reactant. Therefore, N-doped CDs could be the promising materials to develop a simple, fast and cheap glucose detection method. Chitin is a widely available biomass source, and its main current application is in the production of its water-soluble derivative chitosan [33]. Because of its exceptional biological properties (bioactive, biocompatible, and bioresorbable), excellent film forming ability, nontoxicity, high mechanical strength, cheapness and a susceptibility to chemical modifications, chitosan can be used for immobilization of enzymes and constructing the electrochemistry biosensors by cross-linking with enzymes or other substances [34,35].

**Figure 1:**
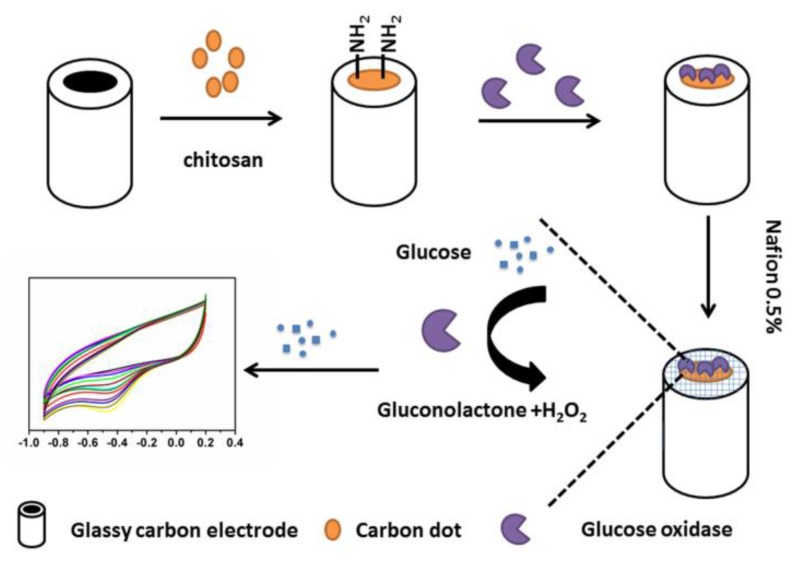
Schematic of the N-doped CDs based electrochemical glucose biosensor.

Recently our group has demonstrated a self-passivized fluorescent N-doped CD which is produced by hydrothermal carbonization of polyacrylamide in one step [36]. The carbonization, surface functionalization and doping occur simultaneously during the hydrothermal treatment, which leads to the formation of the N-doped structure. The resulting N-doped CDs show an excellent electrocatalytic activity in the reduction of O_2_ due to their diatomic side-on adsorption on the N-doped carbon structure. With the one step synthetic N-doped CDs in hand, herein, we construct a novel enzyme immobilization matrix to combine the abovementioned benefits of N-doped CDs and chitosan (Figure 1). The as-prepared composites show obvious electrocatalysis activity toward O_2_. Further, when glucose oxidase (GO_x_) is immobilized on CDs—chitosan composite film, the resulting electrodes demonstrate a favourable linear response to glucose.

## 2. Materials and Methods

Polyacrylamide solution (average M_w_ = 10,000, 50 wt % in H_2_O) and Nafion (product No. 274704) were purchased from Aldrich (Shang Hai, China). All aqueous solutions were prepared with deionized water (18 MΩ·cm^−1^) from a Millipore system. GOx and chitosan were purchased from Alfa Aesar (Shang Hai, China). . D-(+)-Glucose was bought from Sinopharm Chemical Reagent Co., Ltd. (Shang Hai, China). 1-Ethyl-3-[3-dimethylaminopropyl]carbodiimide hydrochloride (EDC) and N-hydroxysuccinimide (NHS) were obtained from J&K Scientific (Shang Hai, China). We used commercial compressed air from a cylinder to maintain the oxygen concentration during electrochemical experiments. All other reagents were analytical grade and used without further purification. All chemicals were stored in a 4 °C refrigerator until used. 

### 2.1. Apparatus

Transmission electron microscopy (TEM) images were taken with a JEM-1011 electron microscope (JEOL, Tokyo,, Japan) at an accelerating voltage of 100 kV. Ultraviolet-visible (UV-vis) absorption of the obtained CNPs solution was carried on a UV-1800(PC) UV-vis spectrophotometer (Mapada, Shang Hai, China). X-ray photoelectron spectroscopy (XPS) analysis was carried on a PHI 5000 Versa-probe X-ray photoelectron spectrometer (JEOL, Tokyo, Japan). All fluorescence spectra of the CDs were measured with a FluoroMax-4 spectrofluorometer (Horiba Scientific, Kyoto, Japan) with a slit width of 5 nm for both excitation and emission.

### 2.2. Preparation and Purification of the N-Doped CDs

In a typical experiment, 8 g of polyacrylamide solution were diluted with 40 mL of deionized water and stirred for 10 min until homogeneous and clear. Then the mixture was transferred to a 100 mL Teflon equipped stainless steel autoclave and sealed. The autoclave was placed in an oven at 260 °C for 24 h to complete the hydrothermal treatment with the heating rate set at 5 °C·min^−1^. When the reaction was complete, the autoclave was cooled down to room temperature. The obtained brown solution without any deposits was neutralized and dialyzed for 3 days (MWCO = 3.5 kD) to precipitate out small molecules. Finally, the yellow solution was freeze dried to obtain the pure N-doped CDs.

### 2.3. Preparation and Purification of the Pure Glucose-Based CDs

In a typical experiment, 2 g of glucose solution were diluted in 20 mL of deionized water and stirred for 10 min until homogeneous and clear. Then the mixture was transferred to a 100 mL Teflon equipped stainless steel autoclave and sealed. The autoclave was placed in an oven at 200 °C for 24 h to complete the hydrothermal treatment at a heating rate of 5 °C·min^−1^. After purified as described for the N-doped CDs, the non N-doped CDs (pure glucose CDs) were obtained.

### 2.4. Preparation of N-Doped CDs/Chitosan/GOx Modified GCE

The GCE was successively polished to a mirror finish using 0.3 and 0.05 μm alumina slurry (Beuhler, Lake Bluff, IL, USA) followed by rinsing thoroughly with double-distilled water. After successive sonication in ethanol and double-distilled water, the electrode was rinsed with double-distilled water and allowed to dry at room temperature. A mixture of 3 mg·mL^−1^ N-doped CDs and 3 mg·mL^−1^ chitosan (3.5 μL) was dropped on the pretreated GCE surface and dried at room temperature to form the N-doped CDs/chitosan modified GCE. Then the as-prepared GCE was immersed into freshly 0.1 M PBS containing 2 mg·mL^−1^ GOx and 3 mg·mL^−1^ EDC reagent (EDC and NHS) for 1 h at 4 °C in refrigerator. The resulting N-doped CDs/chitosan/GOx modified GCE was then rinsed throughout with double-distilled water to wash away any loosely adsorbed enzyme molecules and EDC reagent. To maintain the stability of the modified GCE, a drop of 4.0 μL of 0.5% Nafion solution was cast on the membrane before electrochemical measurements. All enzyme-modified electrodes were stored in 0.1 M PBS (pH 7.0) at a 4 °C in refrigerator before use. 

### 2.5. Detection of Glucose with N-Doped CDs/Chitosan/GOx Modified GCE

Cyclic voltammetric experiments were performed on a CHI 812B electrochemical workstation (CH Instruments Inc., Austin, TX, USA). To maintain the oxygen concentration in the solution, all the electrochemical measurements were carried out in air saturated phosphate buffer solution (PBS, 0.1 M, pH 7.0) containing 10% human blood serum at room temperature (20 ± 2 °C) with a conventional three-electrode cell consisting of a glassy carbon electrode (GCE, 3.0 mm diameter) as working electrode, a saturated calomel electrode (SCE) as reference and platinum wire as counter electrodes.

## 3. Results and Discussion

### 3.1. Characterization of N-Doped CDs

**Figure 2:**
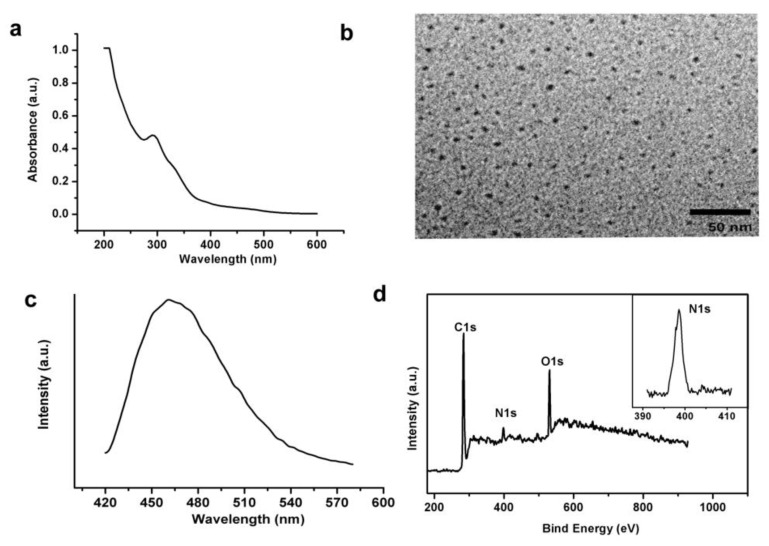
(**a**) UV-vis absorption spectrum of N-doped CDs; (**b**) The typical TEM image of the N-doped CDs; (**c**) PL spectrum of the N-doped CDs with excitation at 280 nm; (**d**) XPS full scan spectrum of the N-doped CDs, inset is XPS N 1s spectrum of the N-doped CDs.

N-doped CDs are synthesized by the method which our group recently reported. Figure 2a displays the UV-vis absorption spectrum of the CDs. The absorption band near 300 nm represents the typical absorption of CDs prepared by hydrothermal carbonization of small molecules containing amide functions. The successful synthesis of N-doped CDs could be observed in the high-resolution transmission electron microscopic (HRTEM) images, in which a uniform size distribution of about 5 nm in diameter is observed (Figure 2b). 

Figure 2c shows the photoluminescence (PL) emission spectrum of the carbon dots with excitation at 400 nm. We can observe a visible emission peak at 480 nm. When the excitation wavelength varies from 340 to 460 nm, the wavelength of the maximum emission would shift from 440 to 520 nm, which is common in fluorescent carbon materials [6]. Figure 2d shows the X-ray photoelectron spectroscopy (XPS) full scan spectrum. It confirms the formation of N-doping in the CDs. The peaks located at 284.5, 398.5, and 531.5 eV correspond to C1s of sp2 C, N1s of the doped N, and O1s of the oxygen functional groups, respectively, and the percentage of atomic N in the sample is about 10.46 wt%. The inset picture of Figure 2d shows the partial XPS spectrum of N1s. The appearance of the N1s peak is postulated to indicate the formation of the nitrogen-containing functional groups during hydrothermal treatment. The hydrothermal treatment forces the surface passivation of N-doped CDs, imparting them with excellent electrochemical properties for immediate biosensing. In summary, luminant N-doped CDs with sizes near 5 nm were successfully synthesized based on hydrothermal treatment of polyacrylamide.

### 3.2. Electrocatalysis of O_2_ Reduction at N-Doped CDs Modified GCE

**Figure 3:**
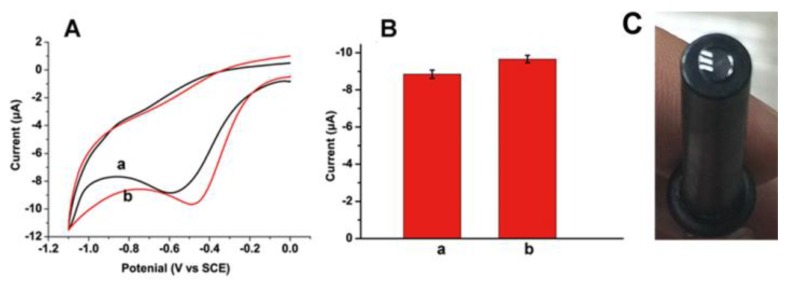
(**A**): Cyclic voltammograms of (**a**) non-N-doped CDs modified electrode and (**b**) N-doped CDs modified electrode in air-saturated 0.1 M pH 7.0 PBS. Scan rate: 100 m·Vs^−1^ (**B**): Electrochemical response for two types of CDs (**a**: non-N-doped CDs and **b**: N-doped CDs); (**C**) the picture of modified electrode.

What is more, we also compared the electrochemical performances of non-N-doped CDs and N-doped CDs in air saturated PBS (Figure 3A). Two different kinds of CDs were prepared by hydrothermal carbonization: non-N-doped CDs from pure glucose (black line of Figure 3A) and N-doped CDs from polyacrylamide (red line of Figure 3A). Both electrodes show a reduction peak near −0.5 V, which is attributed to reduction of oxygen. However, the reduction peak of non-N-doped CDs is at −0.59 V, which is lower than the reduction peak of N-doped CDs at about −0.47 V. What is more, the current of non-N-doped CD_S_ is also lower than that of the N-doped CDs (Figure 3B). These results show that compared to the N-doped CDs from polyacrylamide, the non-N-doped CDs from glucose show less electrocatalysis toward the reduction of O_2_, which is due to the different chemisorption modes of O_2._ On non-N-doped CDs modified electrode, the chemisorption mode of O_2_ is an usual monoatomic end-on adsorption, while, in the N-doped CDs modified electrode, the chemisorption mode of O_2_ changes to a diatomic side-on adsorption, which could effectively weaken the O-O bonding to facilitate the reduction of O_2_ [37]. As a result, we chose the N-doped CDs from polyacrylamide for further application.

### 3.3. Detection of Glucose Based on the N-Doped CDs Modified GCE

**Figure 4:**
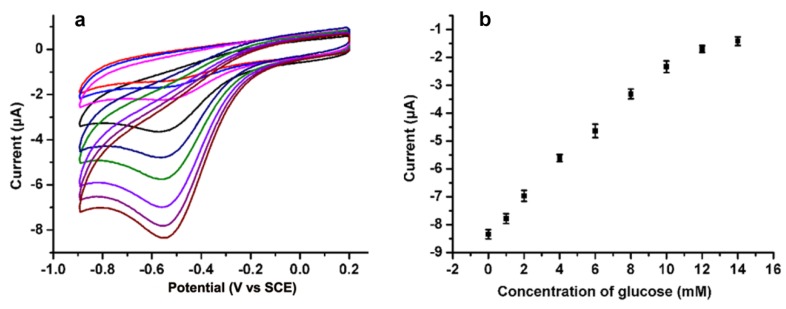
(**a**) Cyclic voltammograms N-doped CDs modified electrode in air-saturated PBS containing 10% of serum sample at a scan rate of 100 mV·s^−1^ with the addition of different concentrations of glucose; (**b**) Electrochemical response curve for different concentration of glucose.

Due to the better electrocatalytical activity of N-doped CDs modified electrodes to O_2_, a N-doped CDs-based biosensor was further developed. The GO_x_ is immobilized into the carbon dot/chitosan nanocomposite matrix prepared through casting the mixed solution containing GO_x_ and EDC reagents for 1 h. The GO_x_ can be directly immobilized on the electrode substrate by amino-carboxyl reactions (Figure 3C). To verify the feasibility of the N-doped CDs-modified biosensor in practical analysis, the biosensor was employed to measure glucose in air-saturated PBS solution containing 10% human blood serum. Figure 4 shows the cyclic voltammograms of the resulting N-doped CDs/chitosan/GO_x_ modified electrode for various concentrations of glucose. With the increase of glucose concentration, the reduction current at negative potential near −0.47 V was decreased. It is well known that the GO_x_-catalysed oxidation of glucose will consume O_2_ and produce H_2_O_2_. The N-doped CDs/chitosan/GO_x_ modified electrode can catalyze the reaction of O_2_, as imagined for a glucose biosensor-based GO_x_ modified electrode (Figure 4). The decrease at negative potential originates from the consumption of O_2_. Although the reduction of produced H_2_O_2_ will result in an increase in the current at negatively applied potentials, this would be entirely counteracted due to the consumption of O_2_. Moreover, the calibration curve corresponding to electrochemical response is linear against the concentrations of glucose over the rangse from 1 to 12 mM (R = 0.99) at −0.47 V. The detection limit of glucose was estimated to be 0.25 mM with a signal-to-noise ratio of 3. This sensitive detection could be attributed to the catalyzed reduction of O_2_ by the N-doped structure and the strong attachment of GO_x_ achieved by the amino-carboxyl reaction. As known, the blood glucose level of a normal person ranges from 4 to 6 mM, so the linear glucose response from 1 to 12 mM based on N-doped CDs/chitosan/GO_x_ modified electrode is suitable for its practical application. This result is better than that using graphene [32], and is still competitive with the result of N-doped CNTs [31]. In addition, compared with other N-doped carbon materials [25,31], it is easier to dope the nitrogen on the carbon materials by using nitrogen-containing raw materials in one step, which makes these N-doped CDs- based glucose biosensors simple and convenient. The successful application in human serum assays indicates that this N-doped CDs based biosensor is promising for the practical application of detecting glucose.

### 3.4. Analytical Performance

**Figure 5:**
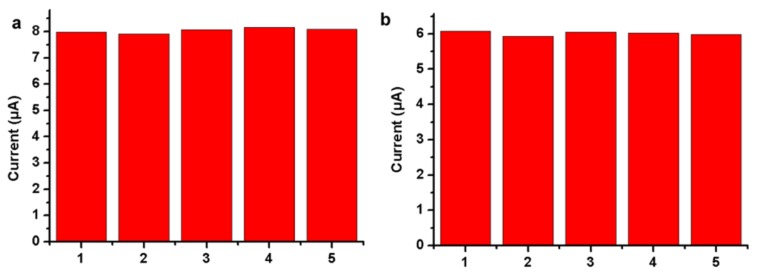
Determination of reproducibility of developed GOx/N-doped CDs/chitosan modified GCE in air-saturated PBS containing 10% of serum sample with: (**a**) 1 mM and (**b**) 5 mM glucose.

The novel biosensor has good reproducibility. We tested the reproducibility in air-saturated PBS containing 10% serum samples (Figure 5). The relative standard deviations (RSD) of the current response to 1 mM (Figure 5a) and 5 mM (Figure 5b) glucose at −0.47 V are 1.23% and 1.04%, respectively, for five successive measurements of different N-doped CDs/chitosan/GOx modified electrodes in air-saturated PBS containing 10% serum samples. The result shows that the designed biosensor has good reproducibility in practical analysis.

**Figure 6:**
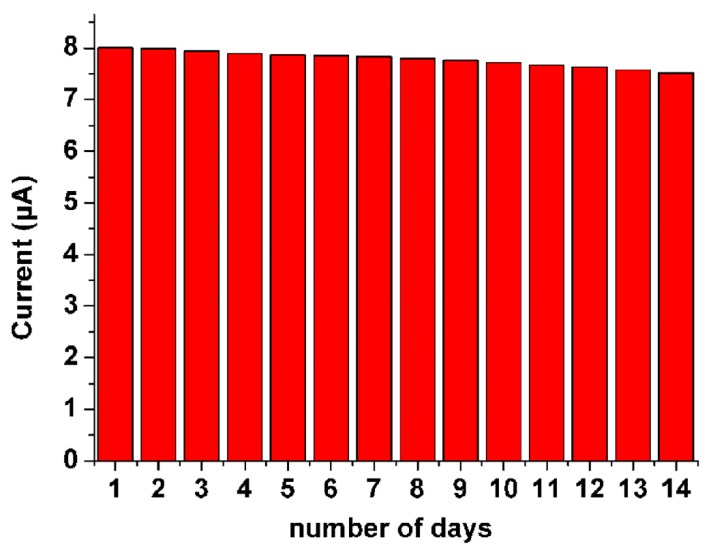
Determination of reproducibility of developed GOx/N-doped CDs/chitosan modified GCE in air-saturated PBS with 1 mM a glucose and in air-saturated PBS containing 10% of serum samples.

The stability of the resulting biosensor was also investigated in air-saturated PBS containing 10% serum samples. The storage stability of the developed N-doped CDs/chitosan/GO_x_ modified electrode is determined from the current response pertaining to the detection of 1 mM glucose each day for a period of 14 days (Figure 6). When stored at room temperature under ambient conditions, there is only a 5.58% decrease in the current response of the developed N-doped CDs/chitosan/GO_x_ modified electrodes for air-saturated PBS containing 10% serum samples. The minor decrease in the functional activity may be due to the spreading of GO_x_ and its conformational change under ambient conditions [32]. This storage stability is good enough for demonstrating the developed laboratory prototype of glucose biosensors under ambient conditions. Compared with other glucose biosensors based on enzymes immobilized on metal nanomaterials [38,39,40], this biosensor shows better storage stability and reproducibility. In addition, compared to other N-doped carbon material-based glucose sensors, this result is also competitive [31,32].

### 3.5. Interference Study

The other important analytical factor for an electrochemical biosensor is the selectivity of the sensor to the target analyte. In this study, interferences of some substances that exist in biological liquids were investigated. The interference tests are carried out by the cyclic voltammograms technique in the presence of 5 mM glucose and the same concentrations of uric acid, ascorbic acid, dopamine and some amino acids such as L-tryptophan, L-tyrosine, and L-cysteine. Compared to the signal change after the addition of pure glucose, there is little signal change after the addition of those interfering substances individually (uric acid 2.32%, ascorbic acid 3.01%, dopamine 3.23%, L-tryptophan 1.54%, L-tyrosine 2.35%, L-cysteine 3.76%). The results show negligible response to the injection of interfering species at their physiological concentration levels, and validate that our N-doped CDs modified biosensors are highly selective towards glucose determination and suitable for the selective glucose determination under physiological conditions.

## 4. Conclusions

A new, simple and cheap glucose biosensor based on enzyme immobilization on N-doped CDs was developed. The high stability, sensitivity and accuracy of this biosensor in the determination of glucose results from the strong attachment of GO_x_ by amino-carboxyl reactions for enzyme immobilization and the nitrogen-induced charge delocalization at N-doped CDs which enhances the electrocatalytic activity toward the reduction of O_2_. It shows great potential application in practical and routine analyses. What is more, the easy and convenient synthesis of N-doped CDs makes the whole experiment fast and cheap, which opens a new promising field of use for N-doped CDs and could also be extended to the immobilization of some other biomolecules.

## Abbreviations

The following abbreviations are used in this manuscript:

CDs	Carbon Dots
GO_x_	Glucose oxidase
N-doped	Nitrogen-doped
